# A Manual of Diseases of the Nose and Throat

**Published:** 1901-08

**Authors:** 


### *BOOK REVIEWS, PAMPHLETS, EXCHANGES.*

---

A Manual of Diseases of the Nose and Throat. By Cornelius Godfrey Coakley, A.M., M.D.; Clinical Professor of Laryngology in the University and Bellevue Hospital Medical College, New York City, etc., etc. Second edition. Revised and Enlarged. Illustrated with 103 Engravings and 4 Colored Plates. Published by Lea Brothers & Co.; pp. 566.

We have carefully read through the bulk of this book, and as a result believe it to be well worth hearty commendation. It is not intended, we presume, as a book of reference, but it succeeds as a short and succinct account of matters of which it treats. We have found nothing specially original or striking in it; it is rather as a safe guide to such as want a not too long-drawn-out treatise on nose and throat operations. There is a satisfactory list of useful prescriptions at the end, and the index is full. The publishers' work has been excellently done. A. W. S.

